# Integrated detrital rutile and detrital zircon ages: a new perspective on the tectonic evolution of South China

**DOI:** 10.1093/nsr/nwae356

**Published:** 2024-10-14

**Authors:** Hao Zou, Hongkui Li, Zhongquan Li, Danlin Wang, Inna Safonova, Huawen Cao, Xin Jin, Haifeng Chen, Changcheng Huang

**Affiliations:** State Key Laboratory of Oil and Gas Reservoir Geology and Exploitation, Chengdu University of Technology, Chengdu 610059, China; College of Earth and Planetary Science, Chengdu University of Technology, Chengdu 610059, China; State Key Laboratory of Oil and Gas Reservoir Geology and Exploitation, Chengdu University of Technology, Chengdu 610059, China; College of Earth and Planetary Science, Chengdu University of Technology, Chengdu 610059, China; State Key Laboratory of Oil and Gas Reservoir Geology and Exploitation, Chengdu University of Technology, Chengdu 610059, China; College of Earth and Planetary Science, Chengdu University of Technology, Chengdu 610059, China; College of Earth and Planetary Science, Chengdu University of Technology, Chengdu 610059, China; Faculty of Geoscience and Engineering, Southwest Jiaotong University, Chengdu 611756, China; Sobolev Institute of Geology and Mineralogy, Novosibirsk 630090, Russia; College of Earth and Planetary Science, Chengdu University of Technology, Chengdu 610059, China; State Key Laboratory of Oil and Gas Reservoir Geology and Exploitation, Chengdu University of Technology, Chengdu 610059, China; College of Earth and Planetary Science, Chengdu University of Technology, Chengdu 610059, China; College of Earth and Planetary Science, Chengdu University of Technology, Chengdu 610059, China

**Keywords:** detrital rutile, detrital zircon, western Yangtze Block, periods of orogeny, provenance

## Abstract

Paleogeographic reconstructions are of key importance for understanding the history of continental breakups and amalgamations during Earth’s history. A special case is the history of the Asian continent, which, compared to other continents, consists of several large cratons and numerous smaller continental blocks. The history of the assembly of South China remains controversial in terms of the timing, Late Neoproterozoic or Early Paleozoic, and the participating continental blocks, e.g. Yangtze, Cathaysia, India and Australia. Detrital rutile U-Pb dating has significant potential with regard to deciphering tectonic settings as rutile frequently crystallizes during orogenic events associated with the processes of collision and subduction. Detrital zircon U-Pb dating is a perfect instrument for identifying the provenance characteristics and age characteristics of sedimentary sources. An integration of these two methods of dating offers better opportunities for reconstructing tectonic settings. This paper presents a first attempt to reconstruct the Neoproterozoic to Early Paleozoic tectonic history and paleogeography of the whole South China based on U-Pb geochronology of rutile and zircon and Hf-in-zircon isotopes from Lower Jurassic Baitianba Formation sedimentary rocks of the western margin of the Yangtze Block, a major part of South China. Our obtained integrated U-Pb rutile and zircon age data show three main age populations of 960–940 Ma, 630–610 Ma and 530–520 Ma. The new U-Pb detrital rutile and zircon ages, compared with former data from Gondwana and Australia, suggest that Yangtze amalgamated with Cathaysia to form South China during the Sibao orogeny at 960–940 Ma. The detrital rutile and zircons of the new 630–610 Ma age group could have been delivered from western Australia during the Late Neoproterozoic to Cambrian Paterson-Petermann orogeny. The abundant 530–520 Ma detrital rutile and zircon ages fit well with the coeval zircon age populations recorded in Gondwana-derived terranes, like India and Indochina.

## Teaser

Using rutile and zircon ages to deduce the tectonic setting of sedimentary basins, this study offers new insights to identify the compressional orogeny or extensional environment of Yangtze Block, South China from Neoproterozoic to Paleozoic.

## INTRODUCTION

Rutile is an important accessory mineral, a rich source of information on the provenance of rocks and is one of the classical minerals used for U-Pb age determination [1]. Rutile can grow in a wide range of P-T (pressure-temperature metamorphic conditions, e.g. [2]) and is sensitive enough to record young metamorphic events that could affect the protolith [3]. Meanwhile, rutile is among the most stable of the detrital minerals in sedimentary systems, and endures sedimentary cycles of transport and diagenesis. It typically grows during collision- and subduction-related orogenies and, to a lesser extent, during extensional processes. Detrital zircon is often used to analyze the source of sediments. Rutile and zircon can record different aspects of crustal recycling and growth: zircon constrains the timing of high-temperature processes, both magmatic and metamorphic, while rutile records episodes of peak metamorphism, e.g. eclogitization, or histories of exhumation and cooling. Crustal movement can be divided into horizontal and vertical movement, and detrital rutile is sensitive to horizontal compression rather than vertical extension [1]. For instance, an overlapping of age peaks of detrital rutile and detrital zircon suggests the presence of a convergent/collisional tectonic setting during this or that period. In the case of clastic sedimentary rocks, we can use detrital rutile and zircon to reconstruct the cycles of transportation and diagenesis of sediments and to discriminate between sedimentary basins formed in convergent/collisional and extensional settings [3].

**Figure 1.**
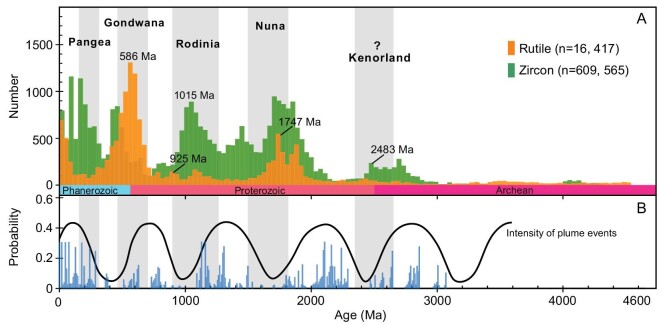
(A) A compilated histogram of globally recorded detrital rutile and detrital zircon U-Pb ages (for the data for detrital rutile see Table S1; the detrital zircon data are from ref [15]). (B) U-Pb zircon age probability plot of the peaks of mantle plume magmatism of large igneous provinces [16]).

Kernel density estimates of global detrital rutile and detrital zircon U-Pb age data show several peaks during Earth's history (Table S1) that match the main periods of the supercontinent assemblies and related orogenies (Fig. 1; [4]). Of special interest are the Grenvillian and Pan-African orogenies that resulted from the amalgamation of supercontinents Rodinia and Gondwana, respectively. These two tectonic events have been well recorded in East Gondwana blocks [5].

South China is typically regarded as an integral part of both Rodinia and Gondwana, but its paleogeographic position remains controversial [6], mostly because it was affected also by the Pan-African orogeny. No consensus has been achieved yet on the assembly and tectonic evolution of South China during the Neoproterozoic to Early Paleozoic transitional period (e.g. [7,8]). A key factor in understanding the detachment of South China from Rodinia and its drift to Gondwana is a probable connection between South China and India (e.g. [9,10]). Traditionally, the relationships between South China and India have been studied in terms of magmatism and sedimentation [10–12]. In particular, several research teams used U-Pb detrital zircon age data from Ediacaran-Cambrian sedimentary formations of South China to understand their provenances [13,14]. However, until recently, samples of clastic rocks have been mainly from eastern South China, that is from Cathaysia, thus leaving their geographic coverage limited [14]. To solve those controversial issues and provide more robust evidence on the paleogeographic position of South China in the mosaic of the Rodinia and Gondwana supercontinents, we combined U-Pb age data from detrital rutile and zircon from clastic rocks of the western Yangtze Block. That allowed us to determine the age of crustal growth (U-Pb igneous zircon ages) and metamorphism (U-Pb rutile ages) and to contribute to a better understanding of the Early Paleozoic history of South China.

## GEOLOGICAL BACKGROUND AND SAMPLING

**Figure 2.**
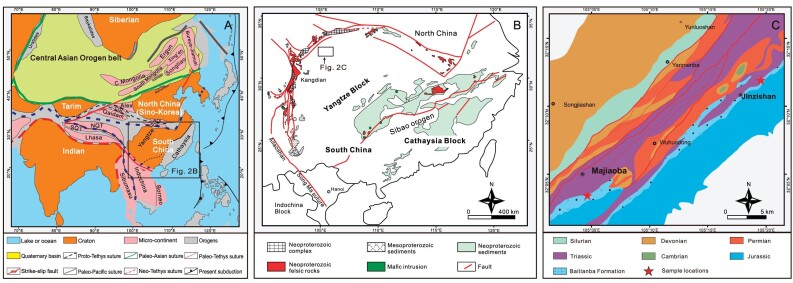
(A) Geotectonic scheme of Asia showing the location of South China (modified from [21,22]). (B) Main tectonic features of South China (modified from [23–25]). (C) A geological sketch of the study area showing the sampling sites in the Baitianba Formation (modified from 1 : 50 000 geological map of Majiaoba).

South China is one of the largest cratons in eastern Eurasia (Fig. 2A). The North China Block is in the north, the Tibetan Plateau is in the west, the Indochina Block is in the southwest and the Pacific Plate is in the east. South China is actually a composite continental block formed by the assembly of the Yangtze (northwestern part) and Cathaysian (southeastern part) blocks amalgamated through the Sibao orogenic belt (Grenvillian orogenic belt; [10,17]). An important part of the Yangtze Block is the Sichuan Basin. The study area is located in the northwestern part of the Sichuan Basin, specifically in the Longmenshan orogenic belt separating the Sichuan and Songpan-Ganze basins (Fig. 2B).

The Yangtze Block is unconformably overlain by Middle to Upper Neoproterozoic and Lower Paleozoic successions [18]. The western part of the region includes Early Neoproterozoic (ca. 1070–750 Ma) volcanic and sedimentary units intruded by felsic plutonic rocks and also by lesser abundant mafic to ultramafic plutons [19]. This study focuses on the Jurassic terrigenous strata of lacustrine origin, the 300 m thick Baitianba Formation, that are exposed in the western margin of the Yangtze Block. The Baitianba sedimentary rocks have unconformable contact with the underlying Early Triassic Xujiahe Fm. and pseudo conformable contact with the overlapping Cretaceous deposits. The lithology of Baitianba Fm. is dominated by arkose sandstone. The Sichuan Basin evolved from an oceanic basin to a continental margin and then to a continental setting during the Late Triassic [20]. Therefore, Sichuan Early Jurassic sedimentary rocks record the cessation of the ocean-continent transition.

**Figure 3.**
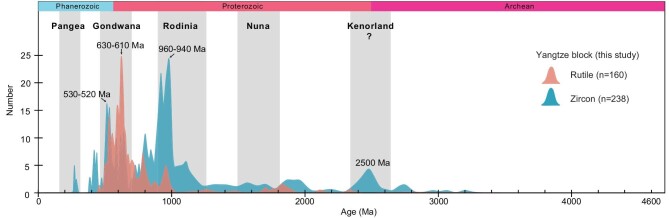
Comparison of U-Pb age curves of detrital rutile and detrital zircons from the Yangtze Block (this study).

A total of 10 kg samples of sedimentary rocks were collected from the western margin of the Yangtze Block for detrital rutile and detrital zircon analyses (samples JZS01 and MJB01; Fig. 2B). Before analyses, the surface was cleaned using diluted HNO_3_ (3%, v/v) and pure alcohol to remove any lead contamination. All rutile and zircon grains were documented with transmitted and reflected light microphotographs as well as cathodoluminescence (CL) images, to select the best domains in grains for analyses (Fig. 3). To make each population statistically representative, detrital rutile and zircon grains of variable sizes and shapes were selected randomly but grains with cracks or inclusions were left out. The results of U-Pb detrital rutile and zircon dating and Lu-Hf zircon isotope analyses are listed in Tables S2, S3 and S4, respectively.

## RESULTS

From a sandstone sample of the western margin of the Yangtze Block, 160 detrital rutile grains and 238 detrital zircon grains were analyzed and are listed in Tables S2 and S3. This rutile and zircon has oscillatory zoning in CL images (Fig. S1) and Th/U values > 0.4 indicating its igneous origin [26]. Zircons of different origins may also exhibit differences in their chondrite-normalized REE (rare earth elements) patterns. Magmatic zircons exhibit relative depletion of light REE, relative enrichment of heavy REE, and obvious left-leaning patterns of Ce positive and Eu negative anomalies [26]. The standardized distribution pattern chart of detrital zircon REE chondrite meteorites in this study indicates that detrital zircons have magmatic zircon characteristics. Most detrital rutile and detrital zircons are transparent to light yellow, with grain sizes varying from 50 to 160 μm. They have a clear ring structure, and the shape of particles is mainly semi-authigenic to irregular short columnar or elliptical. Most rutile and zircon particles are rounded, while a few have obvious edges and corners. A variety of internal zonation exists, ranging from strong oscillatory zoning, to weak zonation. Variations, both in shape and internal structure, suggest that the well-rounded rutile and zircons might have experienced long-distance transport and multistage reworking, and the euhedral grains were likely to have been deposited relatively close to source areas. ^206^Pb/^238^U and ^207^Pb/^206^Pb ages were used for zircons younger and older than 1000 Ma, respectively [27], for plotting using Density Plotter software [28].

### U-Pb detrital rutile ages

The detrital rutile U-Pb isotope ratios were plotted onto a Tera-Wasserburg diagram (Table S2; Fig. 3). The U-Pb ages range from 2142 to 462 Ma to form three main populations at 960–940, 630–610 and 530–520 Ma. In addition, three subordinate peaks are seen at ∼865 Ma, ∼677 Ma and ∼568 Ma. In summary, there are 43 detrital rutile grains distributed in the Early Neoproterozoic, accounting for 27% of the total; 62 detrital rutile grains distributed in the Ediacaran, accounting for 39% of the total; and 20 detrital rutile grains distributed in the Cambrian, accounting for 13% of the total.

### U-Pb detrital zircon ages

The detrital zircons yielded U-Pb ages ranging from 3062 to 271 Ma (Table S3; Fig. 3). The U-Pb data from these concordant grains yield one dominant age population peak at 950 Ma. Secondly, the Early Paleozoic particles accounted for a relatively large proportion, resulting in an age peak at 520 Ma. The Paleoproterozoic grains are fewer in number and show an age peak at 2500 Ma (Fig. 3).

### Lu-Hf zircon isotopes

The ^176^Lu/^177^Hf ratios of the detrital zircon samples are <0.002, with averages of 0.000774 and 0.000841, respectively (Table S4). Lu-Hf isotopes were measured in 128 zircons from this sample. Forty-one zircons yielded positive ε_Hf_(t)values, some showing a crustal incubation time of <300 Ma [29]. The ca. 2500 Ma zircons show ε_Hf_(t) values ranging from −5.21 to +2.49. The 950 and 520 Ma zircons are characterized by wider ranges of ε_Hf_(t) values: −12.08 to +11.6 and −24.20 to +4.48, respectively.

## DISCUSSION

### Periods of orogeny and provenance analyses

**Figure 4.**
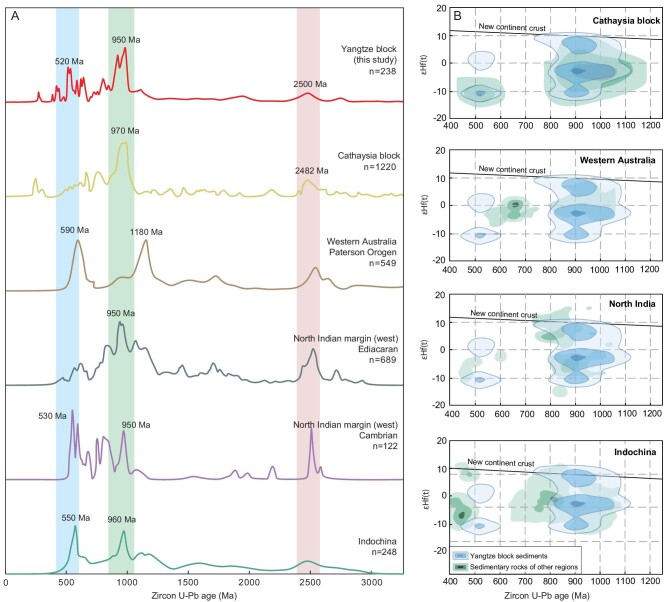
(A) U-Pb age frequency distribution of detrital zircons. Data sources: Yangtze Block (this study); Cathaysia [51]; western Australia [46,52]; India [14]; Indochina [53]. (B) U-Pb age vs. ε_Hf_(t) value-age diagram for detrital zircons from the Yangtze Block (this study); Cathaysia [32,54,68]; western Australia [46,55]; North India [56,57]; Indochina [58,74].

Worldwide coeval orogenic belts are used in reconstructing the assemblies of supercontinents. The Rodinia supercontinent is a global continent that was formed by a continental collision ca. 1000 Ma, and this global orogeny is known as the Grenvillian orogeny. Gondwana is a supercontinent that formed during the Pan-African orogeny from the Late Neoproterozoic to the Early Paleozoic. However, whether South China participated in these two supercontinent collisions has been controversial, and the existence of these two global orogenic belts in the South China block is equivocal. There are no magmatic or metamorphic formations of 650–500 Ma ages in South China to date, indicating a possible external source terrane(s) for the 650–500 Ma components [30]. The source for all the other clastic rocks can be traced within South China. Some researchers think that South China is related to the Pan-African orogeny, namely the assembly of the Gondwana supercontinent [31,32]. Although magmatism related to the Pan-African orogeny rarely occurs in South China, the tectonism is very obvious in the Early Neoproterozoic and with large-scale magmatic activity, especially along the Sibao orogenic belt (Fig. 2B). Most of the detrital zircons investigated in this study have a Th/U ratio higher than 0.1 and show fine-scale oscillatory growth zoning, indicating that the detritus was provided by a magmatic source. Thus, they provide a great means of deciphering the timing of magmatic events and the crustal evolution of the Yangtze Block. Sandstone samples from the western margin of the Yangtze Block contain detrital zircons yielding abundant zircons of 960–940 Ma (Sibao orogeny), plus those of 630–610 Ma (new orogeny?) and 530–520 Ma (Pan-African orogeny; Fig. 3). The data suggest that the source regions possibly experienced three broad episodes of tectono-magmatic events. To compare the provenance of detrital zircons we used the U-Pb zircon ages from the Cathaysia Block, Indochina, Australia and India (Fig. 4).

In this study, the U-Pb age spectra from both detrital rutile and zircon show peaks at 960–940 Ma, sharp peaks at 630–610 Ma and softer peaks at 530–520 Ma (Fig. 3). The U-Pb age spectra of detrital zircons from the western margin of the Yangtze Block are similar to those of the Cathaysia Block, both peaking at 970 and 2480 Ma (Fig. 4). In addition, the age distribution of studied detrital zircons is inconsistent with that of the Yangtze Block, characterized by predominant age groups between 720 Ma and 910 Ma, with a peak at 830 Ma [33]. Therefore, we propose that the Neoproterozoic grains (960–940 Ma) were probably derived from the Sibao orogenic belt. The reason is that the Neoproterozoic zircon grains (960–940 Ma) are moderately rounded suggesting that they could be transported from a source located not far from the Yangtze Block, i.e. the Cathaysia Block.

Recently, 990–950 Ma and 1100 Ma ages have been obtained in the south Cathaysia Block and the southeast of the Yangtze Block, respectively [34]. More evidence for that comes from (i) the Grenvillian zircons hosted by metasediments of Hainan and northern Fujian of the Cathaysia Block [35]; (ii) both negative and positive ε_Hf_(t) values (e.g. Fig. 4; [36]) of ca. 950 Ma detrital zircons of the Yangtze Block (Fig. 4) [36] and 1000–800 Ma zircons of the Cathaysia Precambrian metasedimentary rocks [37]; (iii) voluminous Neoproterozoic arc magmatic rocks (970–830 Ma) and SSZ (supra-subduction zone) type ophiolitic suite are distributed in the Sibao orogen and carry abundant zircons with positive ε_Hf_(t) values and negative ε_Hf_(t) values [38,39].

Further examinations of the 620–400 Ma detrital zircons from the Yangtze Block reveal the following specific features. Most of these zircon particles show complicated crystal morphology and they are oval, indicating that they have undergone long-distance transportation and may come from other blocks. The 620–400 Ma detrital zircon population is a prominent feature of the Gondwana continent [40,41]. Of special interest is the peak at 630–610 Ma, which we fixed out for the first time for South China. The source of these detrital zircons cannot be the Terra Australis orogen, as the orogeny there began at 580 Ma [42]. Some people believe that they may have originated from the East African orogenic belt [43], but the 800 Ma age group of the East African orogenic belt is significantly different from this study (e.g. [44]). Bagas [45] discovered a granite age of 700–600 Ma in western Australia. Therefore, the central Australia segment has been previously considered to be a source of 630–610 Ma detrital zircons (e.g. [46]) as it experienced the Paterson-Petermann orogeny at 650–520 Ma [47]. According to a study by GSWA *et al.* [47], the U-Pb age of Paterson-Petermann orogeny granite is ca. 630 Ma, which is consistent with the peak U-Pb age of detrital zircons in South China (Fig. 4). In addition, the Hf isotopes of granite (ε_Hf_(t) +2 to 5) are also very similar to this study [46]. Our data from detrital zircons, both U-Pb ages and ε_Hf_(t), confirm such a model (Fig. 4). Based on the above analyses, we argue that the amalgamated South China was probably subducting under an active margin of Australia during the Ediacaran to Early Paleozoic [13], and there may have been a potential exchange of the material derived from the provenance formed between the two blocks. The new 630–610 Ma peak from South China (Fig. 3) is close to the ca. 590 Ma age of the zircons from sedimentary rocks of western Australia (Fig. 4). Consequently, we suggest that the Australian active continental margin of Gondwana served as a source of the 630–610 Ma zircons.

After the peak at 630–610 Ma, the detrital zircon age spectra of the Yangtze Block become similar to those of northern India, but different from those of western Australia [14]. Figure 4 illustrates that the 530–520 Ma peak is present in the detrital zircon U-Pb age spectra of both the Yangtze Block and the northern margin of India [49]. The Hf isotope plot shows few, if any, 530–530 Ma zircons in clastic rocks of both northern India and Yangtze, with positive ε_Hf_(t) values [50]. In addition, the age spectrum of the detrital rutiles from the Yangtze Block also shows a peak at 530–520 Ma (Fig. 3). The coinciding age peaks at 530–520 Ma obtained from both detrital zircon and rutile suggest a contemporaneous compressional tectonic event, probably related to the Pan-African orogeny and associated orogenic belts formed during the final assembly of the Gondwana supercontinent [48]. These findings imply that during the assembly of Gondwana, the northern margin of India was closely connected with the Yangtze Block, and served as a source of early Cambrian sediments/zircons in Yangtze.

### The amalgamation of the Yangtze and Cathaysia

The Grenvillian orogeny was widespread around Laurentia and East Antarctica [59]. It resulted in the formation of orogenic belts that amalgamated a series of continental blocks to form the supercontinent Rodinia [60]. The traditional view is that South China, a part of Rodinia, was formed by the amalgamation of the Yangtze Block and Cathaysia Block along the Sibao orogenic belt of the two blocks. However, the timing of the amalgamation remains debatable [61]. One team argues that the Yangtze and Cathaysia blocks were amalgamated in 1000–900 Ma, and the Sibao orogenic belt is part of the global Grenvillian orogeny [35,62]. Another team, using the latest geochronological and geochemical data, suggested that the amalgamation of the two blocks was 820 Ma, or even later, and the Sibao orogenic belt is later than the Grenvillian orogeny [63]. Here we use detrital rutile and detrital zircon age data to limit the merger time of the Yangtze Block and Cathaysia Block.

It has been found that there is a Grenville metamorphic belt at the southern margin of the Yangtze Block [35,64]. Grenvillian granitoids and high-grade metamorphic rocks are absent there. However, granite gneisses and metamorphic gabbros near Panzhihua in the western Yangtze Block [19], dacite in the northwestern Yangtze Block [65] and Neoproterozoic metasediments [12,66], show zircons with Grenvillian ages. These data suggest that Grenvillian high-grade metamorphism propagated from the northern to the western margins of the Yangtze Block [67]. The 960–940 Ma detrital rutile and zircon ages probably reflect the Grenvillian orogeny being linked to the assembly of the Rodinia supercontinent [9]. The Sibao orogenic belt was thought to be a typical Grenvillian orogenic belt [34,68], a collision between the Yangtze and Cathaysia blocks as a result of the Sibao orogeny, as was postulated in [64]. These Neoproterozoic zircons are believed to be related to the building of the orogenic belt. Secondly, abundant subduction-related magmatic activity at 1000–860 Ma is evident along the Sibao orogenic belt, including the ophiolites and their related igneous rocks [69] and the arc-related volcanic rocks (Fig. 2B) [70], but no metamorphic rocks younger than 1000 Ma. Finally, after 850 Ma, South China as a whole experienced extension [61]. The Hf-in-zircon isotope data from the western Yangtze Block show a rapid increase in the values of ε_Hf_(t) at 960–940 Ma, which is consistent with the ages of zircons from Neoproterozoic formations of the central Yangtze Block [71,72]. Therefore, a large amount of mantle material was added to the crust during that period, i.e. during the assemblage of the Rodinia supercontinent. All these data prove that the Yangtze Block and Cathaysia Block assembled to form the single South China continent at 960–940 Ma.

### Neoproterozoic–Early Paleozoic evolution of South China

The Yangtze, Cathaysia, India and Indochina blocks all show common peaks at 1050–900 Ma (Grenvillian orogeny) and 600–520 Ma (Pan-African orogeny) (Fig. 4). Moreover, the 1000–900 Ma detrital zircons of the Yangtze Block have a wide range of ε_Hf_(t) values (−12.08 to +11.6). This age population is significantly different from the Grenvillian orogeny at 1300–1100 Ma in southwestern Australia [9]. The detrital zircon from western Australia shows a Grenvillian peak of ∼1200 Ma, which does not appear in the Yangtze Block (Fig. 4). So according to the comparative age model of detrital zircon, South China was unlikely to be located near western Australia in the Early Neoproterozoic.

Detrital rutile and detrital zircon in western South China have an obvious peak during the Ediacaran to the Cambrian (Fig. 4). However, South China lacks Ediacaran magmatic, metamorphic and intense deformational records and therefore hardly experienced extensive orogeny during this period [73]. The major age peak at 630–610 Ma and the significant age peak at 530–520 Ma in the sediments may be related to an orogenic event formed by the amalgamation of the Gondwana, although coeval magmatic rocks are rare in South China. The unique 630–610 Ma detrital zircons, which could be provided by the Australian active continental margin, and the first rutile data both suggest a new event of collision orogeny in South China. It is suggested that the 630–610 Ma orogeny could be a result of the collision between South China and Australia.

During the Early Paleozoic, northern India and its surrounding regions experienced two significant orogenic events: the Northern Indian orogeny (530–470 Ma) and the East African orogeny (570–520 Ma) [10]. The coeval zircons produced by these orogenies are believed to be the source of the rounded detrital zircons of the data set under consideration. Those orogenic belts were uplifted during the Neoproterozoic and supplied detrital zircons to the Tethyan Himalaya [8]. The 520 Ma detrital zircons from the Yangtze Block fit, in terms of age, those from northwestern India. The similar spectra of U-Pb ages and distributions of the ε_Hf_(t) values of detrital zircons suggest a link between the South China Block and India [74]. Jiang *et al.* [11] compared the stratigraphic records of the Yangtze Block (South China) and India, and the strata between them are also very similar.

These changes indicate that India collided with South China at ∼530–520 Ma and supplied zircons to the provenance. The ca. 520 Ma detrital zircons that could have been delivered from the north India margin changed dramatically during the Cambrian (Fig. 3). Therefore, we think that South China was located in northern India in the Early Paleozoic [8], not Australia in Gondwana [10,75]. This is another convincing piece of evidence regarding the affinity between South China and Gondwana: comparison of crustal growth histories in zircon source areas. The Hf isotope composition of detrital zircons from samples in this study, as well as from western Australia, northwestern India and the Indochina blocks, indicates that the origin of the Pan-African and Grenvillian zircons is juvenile magmas, the melting of pre-existing crustal rocks, and the reworked character of most pre-Grenvillian zircons. These features show the similarities in the crustal growth histories of the source areas of the Grenvillian and Pan-African zircons recorded in the India and Indochina blocks.

**Figure 5.**
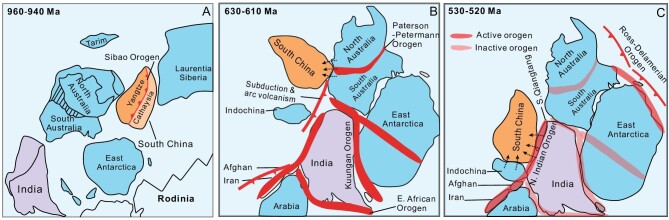
Paleogeographic reconstructions showing position of South China (modified from refs [53]).

The Paleogeography of South China is more controversial [76,77]. Cocks and Torsvik [78], in creating a paleogeographic reconstruction of East Asia, posited that South China and Indochina were a single continent, and this structure is not only consistent with the similarity of detrital zircon age models of Cathaysia, Yangtze and Indochina, but can also connect South China and northern India through Indochina. According to Li *et al.* [79], the paleolatitude of South China during the Ordovician was somewhere between 9° and 24° south, which is consistent with the paleoposition of South China proposed by Metcalfe [80] and with reconstructions based on age spectra of detrital zircons [8]. Based on this, we put forward a paleogeographic reconstruction of South China from the Neoproterozoic to Cambrian (Fig. 5). In the Early Neoproterozoic, South China was formed by the combination of the Yangtze and Cathaysia blocks through the Sibao orogeny, drifted away from the central part of Rodinia during supercontinent breakup, then collided with central Australia in the Early Ediacaran period, and finally collided with northwest India as part of the Gondwana continental combination in the Cambrian period, resulting in the formation of the ‘Pan-African’ orogenic belt along the Himalayas on the northern edge of India. This paleogeographic model is also consistent with the affinity between Early Paleozoic shallow marine fauna of South China and that of eastern Gondwana [81].

## CONCLUSIONS

- The integrated age pattern of detrital rutile and detrital zircons from clastic rocks of the Lower Jurassic Baitianba Formation of South China shows three events of orogeny at 960–940 Ma, 630–610 Ma and 530–520 Ma. The events of the 630–610 Ma orogeny were discovered for the first time.
- Our evidence confirms previous ideas about the formation of South China by the amalgamation of the Yangtze and Cathaysia blocks during the Sibao orogeny, i.e. at ca. 960–940 Ma.
- At 630–610 Ma, South China collided with western Australia, and then collided with India during the final assembly of the Gondwana supercontinent at 530–520 Ma.

## Supplementary Material

nwae356_Supplemental_Files

## Data Availability

The data that support the findings of this study are available from the corresponding author upon reasonable request. All data from this study are available in the online content of this paper and from the Open Science Framework (https://osf.io/hfc2a/?view_only=aaffe4079d8c4e6094ca3456f3e5bd64).
