# Supplementary material for: Integrated detrital rutile and detrital zircon ages: a new perspective on the tectonic evolution of South China
Source: Natl Sci Rev. 2024 Oct 14;11(12):nwae356. doi: 10.1093/nsr/nwae356 (PMC11604069; doi:10.1093/nsr/nwae356)
Supplement: nwae356_Supplemental_Files [file nwae356_supplemental_files.zip › supplementary text.docx]

Supplementary Data

**Integrated detrital rutile and detrital zircon ages: a new perspective on the tectonic evolution of South China**

Hao Zou^1,2^, Hongkui Li^1,2*^, Zhongquan Li^1,2^, Danlin Wang^2^, Inna Safonova^3,4^, Huawen Cao^2^, Xin Jin^1^, Haifeng Chen^2^, Changcheng Huang^2^

*^1^ State Key Laboratory of Oil and Gas Reservoir Geology and Exploitation, Chengdu University of Technology, Chengdu 610059, China*

*^2^ College of Earth and Planetary Science, Chengdu University of Technology, Chengdu 610059, China*

*^3^ Faculty of Geoscience and Engineering, Southwest Jiaotong University, Chengdu 611756, China*

*^4^ Sobolev Institute of Geology and Mineralogy, Novosibirsk 630090, Russia*

*****Corresponding author: Dr. Li Hongkui, email [lihongkui08@cdut.edu.cn](mailto:lihongkui08@cdut.edu.cn)

**This file includes:**

Supplementary Texts: Materials and methods

Supplementary Tables 1-4

References

**Supplementary Texts: Materials and methods**

*Rutile and zircon U-Pb dating*

Samples were crushed and sieved. After being washed and dried, rutile and zircon crystals of different grain sizes were separated by magnetic separation and heavy liquid [1]. Then, rutile and zircon crystals were selected under binoculars for each sample and mounted in epoxy resin. The data in this study were acquired using an Analytikjena PlasmaQuant MS quadrupole ICP-MS equipped with a 193 nm NWR193 Ar-F excimer laser in the Yanduzhongshi Geological Analysis Laboratories Ltd. Rutile standard R13 (SIMS age of 504±4 Ma; [2]) was used as a primary standard to calibrate U-Pb isotope ratios. Each analysis on the rutile began with a 15 second blank gas measurement followed by a further 40 seconds of analysis time when the laser was switched on. Using the laser at 5 Hz and a density of approximately 4 J/cm^2^. A flow of He carrier gas at a rate of 0.6 litres/minute carried particles ablated by the laser out of the chamber to be mixed with Ar gas and carried to the plasma torch. Instrument drift, mass bias and fractionation of the U-Pb ratio are corrected with a standard-sample bracketing method. The final age results of a single sample are plotted in the Tera-Wasserburg diagram without ordinary Pb correction showing intersections and providing the confidence level of 95%.

Zircon detection was completed in Wuhan Sample Solution Analytical Technology Co., Ltd. The main inspection equipment is Agilent Inductively Coupled Plasma Mass Spectrometer (Agilent 7900). All the analyses were determined by using 193nmArF excimer laser to homogenize the beam with a spot diameter of 32 μm, a repetition frequency of 5 Hz and an energy of 10-15 J/cm^2^. The typical ablation time for each analysis is 40 s, and in pits of 30-40 μm depth. Raw count rates for ^206^Pb, ^207^Pb, ^208^Pb, ^232^Th and ^238^U were collected for age determination. The international zircon standard 91500 is used as the external standard for analyzing U-Pb isotopes [3]. The error of a single data point is 2σ. The weighted age average of the samples was calculated by Isoplot/Ex (3.0) software [4], and the zircon U-Pb age concordia diagrams were drawn.

*Zircon Lu-Hf isotope analyses*

One hundred and twenty-eight representative detrital zircons were selected from samples with known U-Pb ages for Lu-Hf isotope in situ analyses at Wuhan Sample Solution Analytical Technology Co., Ltd. (WHSSATC), Hubei, China, by using a Neptune Plus MC-IC-MS equipped with a Geolas HD 193 nm excimer ArF laser. The spot size and frequency of the laser were set to 44 μm and 6 Hz, respectively. The energy density of the laser ablation was 10 J/cm^2^. The ablation time was 40s for each measurement to make pits 30-40 μm deep. Zircon 91500 and GJ-1 were the external standards giving weighted average ^176^Hf/^177^Hf ratios of 0.282261 ± 0.000022 and 0.282005 ± 0.000007, respectively [5].

**Supplementary Tables**

Table S1. U-Pb dating data of detrital rutile from earth history

Table S2. U-Pb dating data of detrital rutile from the western margin of Yangtze Block

Table S3. U-Pb dating data of detrital zircon from the western margin of Yangtze Block

Table S4. Hf-isotope data of detrital zircons from the western margin of Yangtze Block

These Tables are available at NSR online and https://osf.io/j7dvw/?view_only=bccd55df41c34e018c30a562cc3a6e27

**References**

1. Mange M A, Maurer H F W. Heavy minerals in colour. *London: Chapman and Hall* 1992; **147** p.
2. Schmitt A K, Zack T. High-sensitivity U-Pb rutile dating by secondary ion mass spectrometry (SIMS) with an O2+ primary beam. *Chemical Geology* 2012; **332**, 65–73.
3. Wiedenbeck M et al. 2004, Further characterisation of the 91500 zircon crystal. *Geostandards and Geoanalytical Research* 2004; **28** (1), 9-39.
4. Ludwing K R. Isoplot: A Geochronological Toolkit for Microsoft Excel: Geochronology Center, Special Publication. 2003; pp. 1-70.
5. Griffin W L, Powell W J, Pearson N J, O' Reilly S Y. GLITTER: data reduction software for laser ablation ICP-MS. *Mineralogical Association of Canada* 2008; **308**-311.
